# Supplementary material for: Actomyosin organelle functions of SPIRE actin nucleators precede animal evolution
Source: Commun Biol. 2024 Jul 8;7:832. doi: 10.1038/s42003-024-06458-1 (PMC11231147; doi:10.1038/s42003-024-06458-1)

Supplementary Table 1. Prokaryotic and eukaryotic protein expression vectors used in this study.

Construct	Plasmid	Cloning	Purpose
GFP-Mb-SPIRE	pAcGFP1-C1-Mb-SPIRE	aa 2-522 (*Xho*I / *Kpn*I)	colocalisation GST-pulldown
	pENTR-EGFPC1-Myc-Mb-SPIRE	aa 2-522	melanosome rescue
GFP-Mb-SPIRE-KW	pAcGFP1-C1-Mb-SPIRE-KW	aa 2-272 (*Kpn*I / *Bam*HI)	GST-pulldown
GFP-Mb-SPIRE-ΔKW	pAcGFP1-C1-Mb-SPIRE-ΔKW	aa 273-522 (*Kpn*I / *Bam*HI)	colocalisation GST-pulldown
GFP-Mb-SPIRE-aa2-289	pAcGFP1-C1-Mb-SPIRE-aa2-289	aa 2-289 (*Kpn*I / *Bam*HI)	GST-pulldown
GFP-Mb-SPIRE-aa2-324	pAcGFP1-C1-Mb-SPIRE-aa2-324	aa 2-324 (*Kpn*I / *Bam*HI)	GST-pulldown
GFP-Mb-SPIRE-aa290-522	pAcGFP1-C1-Mb-SPIRE-aa290-522	aa 290-522 (*Kpn*I / *Bam*HI)	GST-pulldown
GFP-Mb-SPIRE-aa325-522	pAcGFP1-C1-Mb-SPIRE-aa325-522	aa 325-522 (*Kpn*I / *Bam*HI)	GST-pulldown
GFP-Mb-FMN-FH2-FSI	pAcGFP1-C1-Mb-FMN-FH2-FSI	aa 1116-1593 (*Eco*RI / *Kpn*I)	GST-pulldown
GFP-Hs-SPIRE1	pENTR-EGFPC1-Myc-Hs-SPIRE1	aa 2-742	melanosome rescue
GFP-Hs-SPIRE2	pENTR-EGFPC1-Myc-Hs-SPIRE2	aa 2-714	melanosome rescue
GFP-Mm-FMN1-FH2-FSI	pAcGFP1-C1-Mm-FMN1-FH2-FSI	aa 969-1466 (*Eco*RI / *Bam*HI)	GST-pulldown
GFP-Mm-FMN2-FH2-FSI	pAcGFP1-C1-Mm-FMN2-FH2-FSI	aa 1135-1578 (*Kpn*I / *Xba*I)	GST-pulldown
GFP-Sr-SPIRE	pAcGFP1-C1-Sr-SPIRE	aa 2-683 (*Eco*RI / *Bam*HI)	GST-pulldown
GFP-Sr-SPIRE-ΔKW	pAcGFP1-C1-Sr-SPIRE-ΔKW	aa 269-683 (*Eco*RI / *Bam*HI)	GST-pulldown
GFP-Sr-SPIRE-aa2-371	pAcGFP1-C1-Sr-SPIRE-aa2-371	aa 2-371 (*Eco*RI / *Bam*HI)	GST-pulldown
GFP-Sr-SPIRE-aa2-401	pAcGFP1-C1-Sr-SPIRE-aa2-401	aa 2-401 (*Eco*RI / *Bam*HI)	GST-pulldown
GFP-Sr-SPIRE-aa2-405	pAcGFP1-C1-Sr-SPIRE-aa2-405	aa 2-405 (*Eco*RI / *Bam*HI)	GST-pulldown
GFP-Sr-SPIRE-aa372-683	pAcGFP1-C1-Sr-SPIRE-aa372-683	aa 372-683 (*Eco*RI / *Bam*HI)	GST-pulldown
GFP-Sr-SPIRE-aa384-683	pAcGFP1-C1-Sr-SPIRE-aa384-683	aa 384-683 (*Eco*RI / *Bam*HI)	GST-pulldown
GFP-Sr-SPIRE-aa406-683	pAcGFP1-C1-Sr-SPIRE-aa406-683	aa 406-683 (*Eco*RI / *Bam*HI)	GST-pulldown
Sr-MYO5 (Choanoflagellate expression)	NK644-mNeonGreen-Sr-MYO5-cc-GTD	*see methods section for details*	Choanoflagellate Live cell imaging
Sr-SPIRE (Choanoflagellate expression)	NK644-mScarlet-I-Sr-SPIRE	*see methods section for details*	Choanoflagellate Live cell imaging
GFP-SPIRE2-ΔKW	pAcGFP1-C1-Hs-SPIRE2-ΔKW	aa 361-714 (*Bam*HI / *Kpn*I)	colocalisation
mRuby3-Mb-FMN-FH2-FSI	pmRuby3-C1-Mb-FMN-FH2-FSI *	aa 1116-1593 (*Eco*RI / *Kpn*I)	colocalisation
mRuby3-Mb-MYO5-GTD	pmRuby3-C1-Mb-MYO5-GTD	aa 1371-1741 (*Kpn*I / *Bam*HI)	colocalisation

mRuby3-Mb-RAB8	pmRuby3-C1-Mb-RAB8	aa 2-204 (*Kpn*I / *Bam*HI)	colocalisation
GST-Mb-MYO5-GTD	pGEX-4T1-NTEV-Mb-MYO5-GTD	aa 1371-1741 (*Bam*HI / *Eco*RI)	GST-pulldown
GST-Mb-RAB8-Q67L	pGEX-4T1-NTEV-Mb-RAB8-Q67L	aa 2-204 (*Bam*HI / *Eco*RI)	GST-pulldown
GST-Mb-RAB8-T22N	pGEX-4T1-NTEV-Mb-RAB8-T22N	aa 2-204 (*Bam*HI / *Eco*RI)	GST-pulldown
GST-Mb-FMN-eFSI	pGEX-4T1-NTEV-Mb-FMN-eFSI	aa 1533-1593 (*Bam*HI / *Eco*RI)	GST-pulldown
GST-Mb-SPIRE-KIND	pGEX-4T1-NTEV-Mb-SPIRE-KIND	aa 5-208 (*Bam*HI / *Eco*RI)	GST-pulldown
GST-Hs-SPIRE1-KIND	pGEX-4T3-Hs-SPIRE1-KIND	aa 2-271 (*Bam*HI / *Sal*I)	GST-pulldown
GST-Sr-MYO5-GTD	pGEX-4T1-NTEV-Sr-MYO5-GTD	aa 1548-1921 (*Bam*HI / *Eco*RI)	GST-pulldown
SNAP-Mm-SPIRE1-KW	pGEX-6P1-SNAP-Mm-SPIRE1-KW	aa 2-433 (*Bam*HI / *Eco*RI)	Actin assembly / disassembly assays
SNAP-Mm-SPIRE1-KW-ΔB,C	pGEX-6P1-SNAP-Mm-SPIRE1-KW-ΔB,C	aa 2-433 deletion of Pro285-Thr349 (*Bam*HI / *Eco*RI) *see methods section for details*	Actin assembly / disassembly assays
SNAP-Mb-SPIRE-KW	pGEX-6P1-SNAP-Mb-SPIRE-KW	aa 2-289 (*Bam*HI / *Eco*RI)	Actin assembly / disassembly assays

Supplementary Figure 1

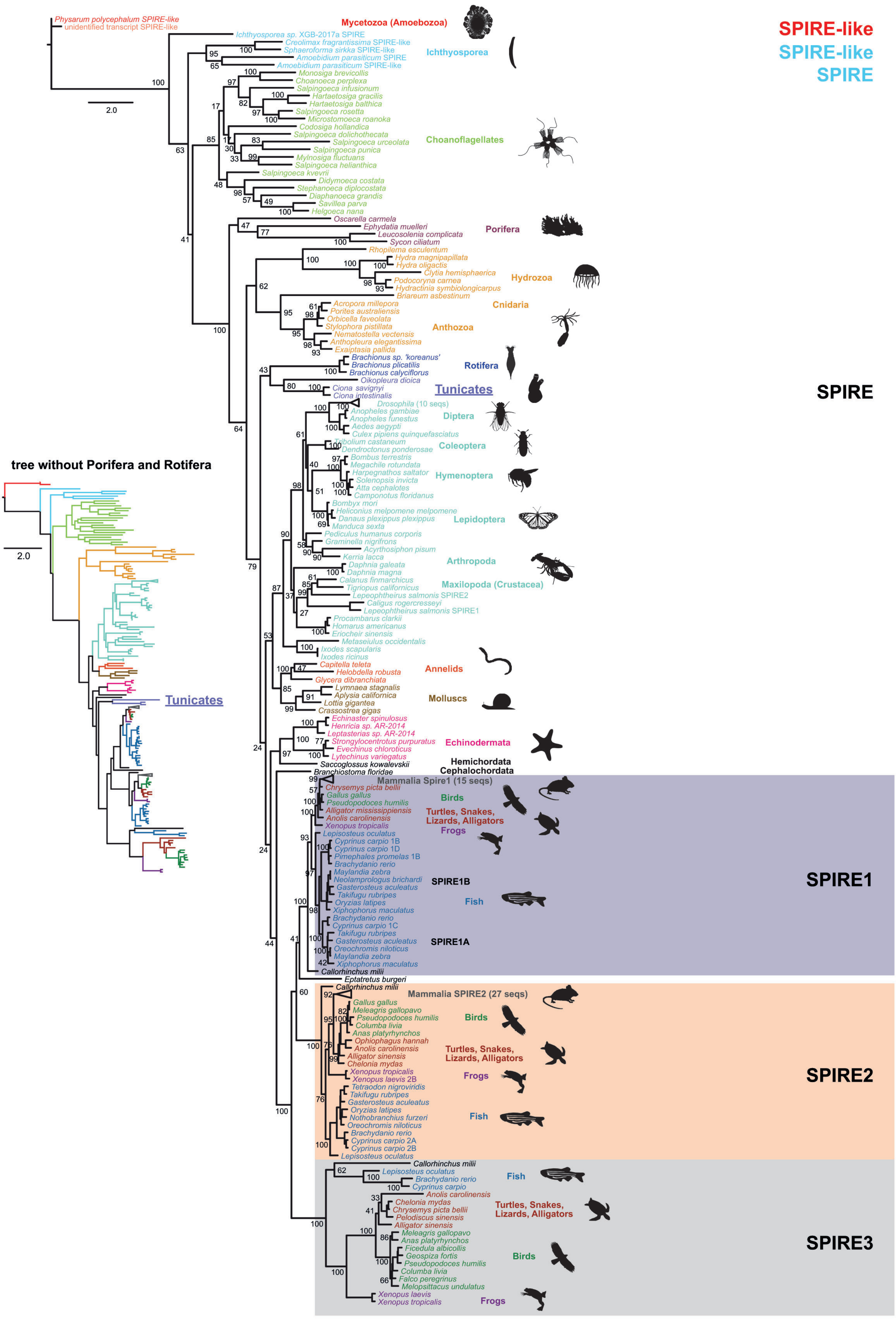

Supplementary Figure 1. SPIRE phylogeny. Phylogeny generated by IQ-TREE (Nguyen et al., 2015) of a CD-Hit-reduced (Li and Godzik, 2006) set (90% identity level) of 217 SPIRE proteins. Support values for the major branches are given as percent bootstrap replicates. For illustrative purposes, the sub-trees of the mammalian and *Drosophila* species SPIRE proteins have been collapsed, with numbers in brackets indicating the number of sequences. Some bootstrap values on short branches were removed for clarity. In this tree, the SPIRE of the Tunicata group with those of the Rotifera outside the other bilaterian SPIRE. The tree in the box was generated with FastTree (Price et al., 2010) based on the same data as the IQ-TREE (Nguyen et al., 2015) tree, except for the four Porifera and three Rotifera SPIRE, which were removed. In this tree, the SPIRE of the Tunicata group sister to the SPIRE of the Vertebrata as expected from their species phylogeny. The different placement of the tunicate SPIRE in dependence of the presence or absence of the Porifera and Rotifera SPIRE sequences suggests branch attraction effects between these sequences. The colouring of the branches is identical to the colour of species names in the large tree. Animal icons were obtained from the PhyloPic website (https://www.phylopic.org/).

Supplementary Figure 2

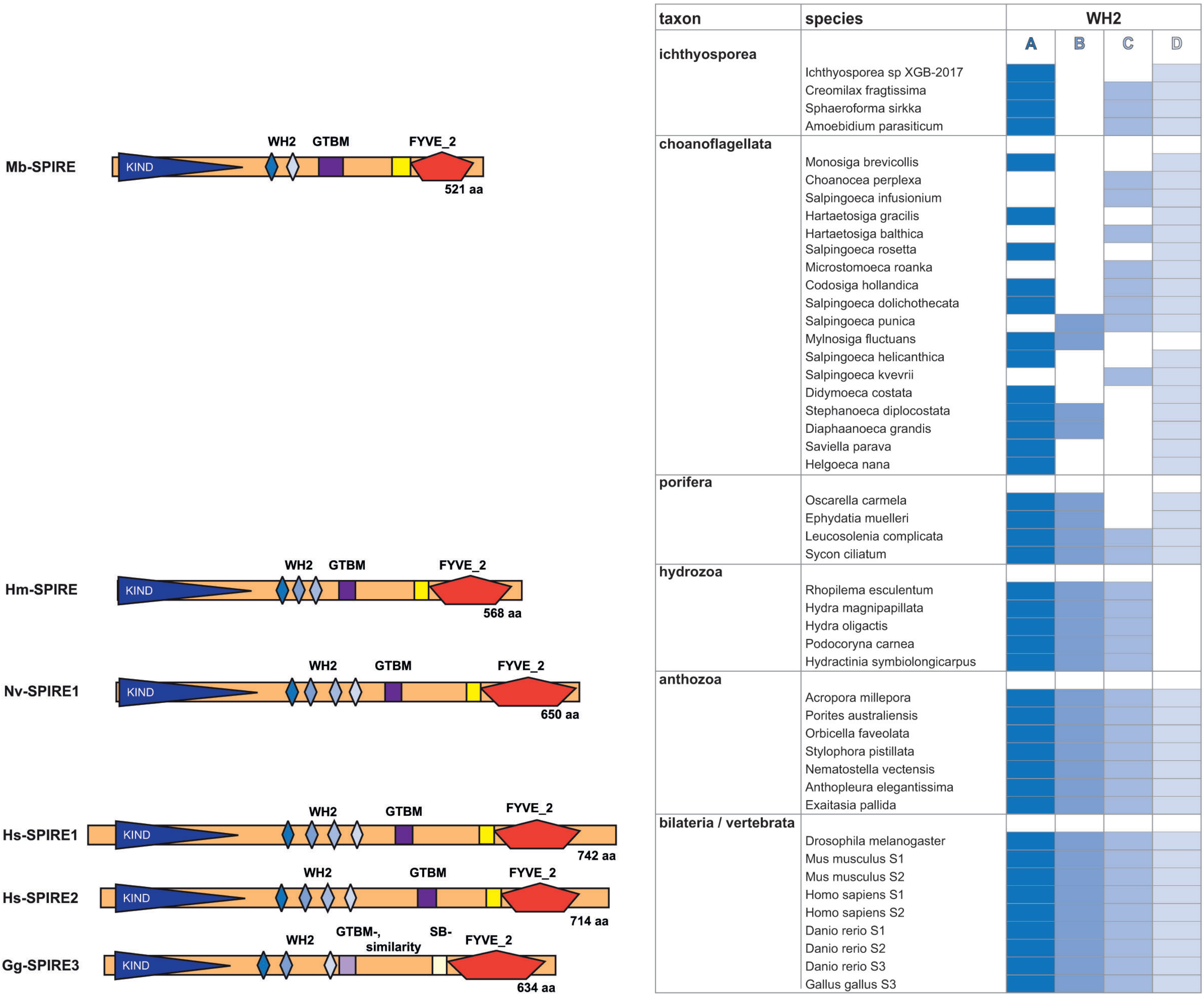

Supplementary Figure 2. SPIRE tandem WH2 domain arrays of extant species. Both, the number and the composition of the individual WH2 domain repeats, vary in the different phylogenetic groups. Protein sequences of WH2 clusters of the indicated species have been aligned and are schematically presented according to their homology to the vertebrate WH2-A, -B, -C, -D domains, respectively. Domain organisations of choanoflagellate, cnidarian and vertebrate SPIRE proteins are shown. Species abbreviations are: Mb, *Monosiga brevicollis*; Hm, *Hydra magnipapillata*; Nv, *Nematostella vectensis*; Hs, *Homo sapiens*; Gg, *Gallus gallus*. KIND, kinase non-catalytic C-lobe domain; WH2, Wiskott-Aldrich syndrome protein homology 2; GTBM, globular tail domain binding motif; FYVE, after Fab 1/YOTB/Vac 1/EEA1.

Supplementary Figure 3

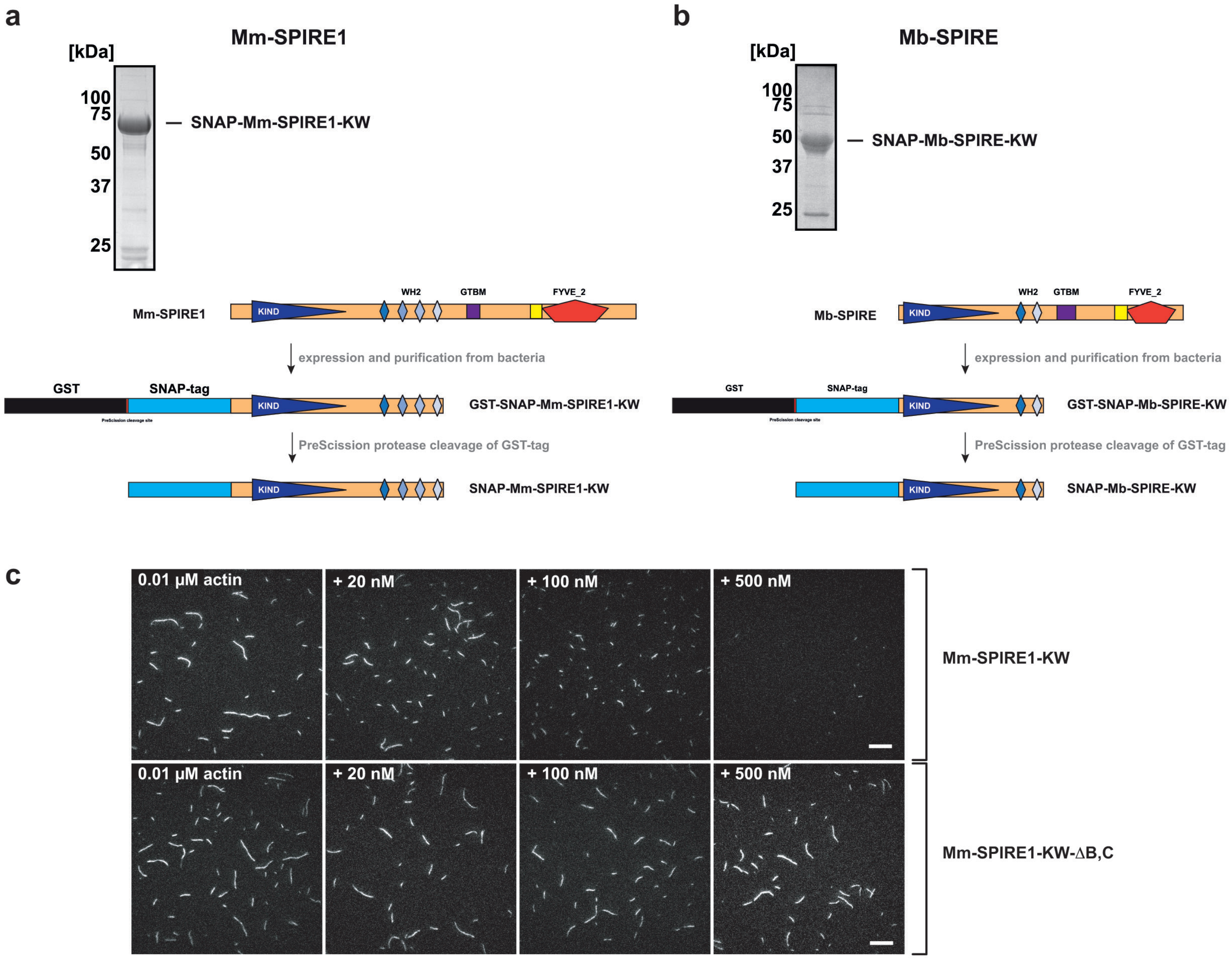

Supplementary Figure 3. Schematic representation of the generation and the final protein purity of SNAP-Mm-SPIRE1-KW (**a**) and SNAP-Mb-SPIRE-KW (**b**) proteins. 10 µg of each protein were run on an SDS polyacrylamide gel and Coomassie stained. (**c**) SPIRE1-KW promotes the disassembly of preformed actin filaments. TIRF microscopy images of actin disassembly (0.01 µM, 20% ATTO488 labelled) in absence or presence of Mm-SPIRE1-KW or mutant Mm-SPIRE1-KW-ΔB,C at the concentrations as indicated. The stills represent the first frame recorded. Scale bar represents 10 µm.

Supplementary Figure 4

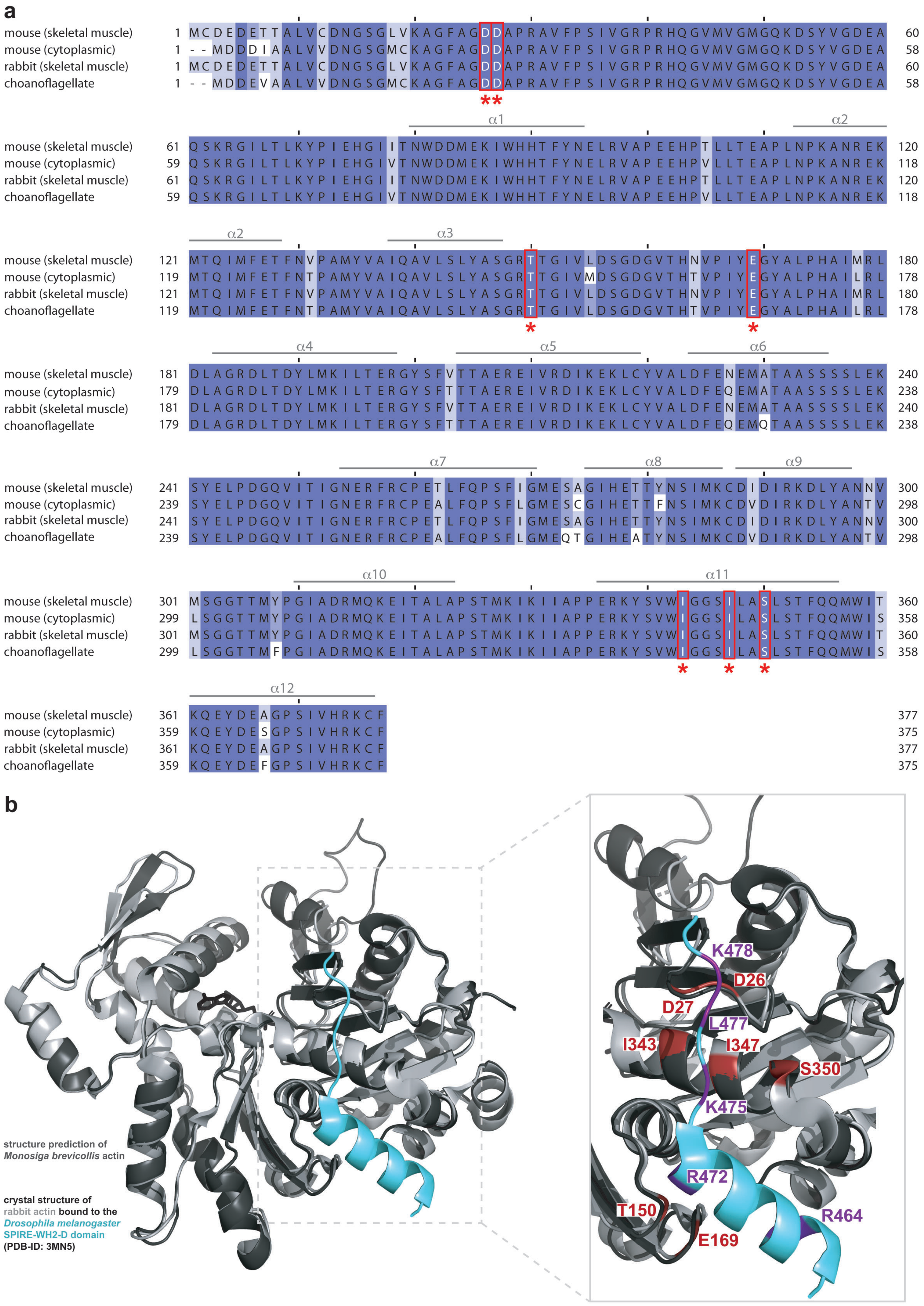

Supplementary Figure 4. Comparison of vertebrate and choanoflagellate actin. (**a**) Multiple protein sequence alignment of actin proteins. The alignment includes mouse skeletal muscle actin (*Mus musculus*; Acta1, NP_001258970.1), mouse cytoplasmic actin (*Mus musculus*; Actb, NP_031419.1), rabbit skeletal muscle actin (*Oryctolagus cuniculus;* Acta1, XP_002722940.2) and choanoflagellate actin (*Monosiga brevicollis;* AAK27412.1). α-helices are labelled (α1-α12) and coloring indicates amino acid conservation at specific positions ranging from high (dark blue) to low (light blue). Actin amino acid residues shown to be involved in actin binding to *Drosophila melanogaster* SPIRE-WH2-D (compare with (b)) are labelled in red and are conserved in choanoflagellates. Numbering indicates amino acid residues. (**b**) Protein structure alignment of the ESMFold (Lin et al., 2023) predicted *Monosiga brevicollis* actin monomer (black) and the crystal structure of rabbit actin (grey) bound to the *Drosophila melanogaster* SPIRE-WH2-D (light blue) sequence (PDB-ID: 3MN5; (Ducka et al., 2010)). The right panel shows a more detailed view on the interaction surface. Rabbit actin and Drosophila WH2 interacting residues are labelled in red and purple, respectively.

Supplementary Figure 5

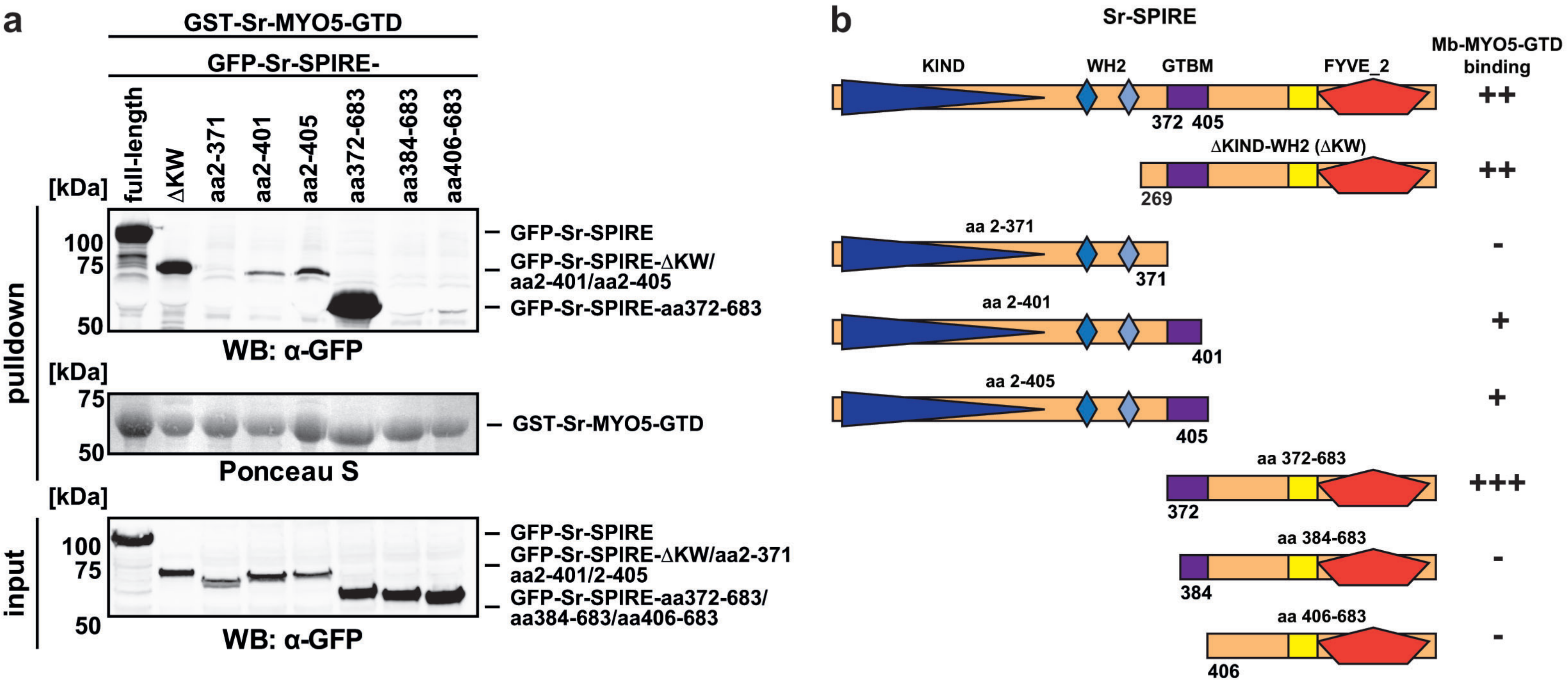

Supplementary Figure 5. Characterisation of the *Salpingoeca rosetta* (Sr) SPIRE MYO5 globular tail domain binding motif. (**a**) GST-pulldown assay with purified GST-Sr-MYO5-GTD from lysates of HEK 293 cells transiently expressing N-terminal and C-terminal AcGFP1-tagged Sr-SPIRE (GFP-Sr-SPIRE) protein fragments. Ponceau S staining shows equal amounts of GST-Sr-MYO5-GTD proteins. N = 2 experimental repeats. KW, KIND-WH2. Numbering indicates amino acid residues. (**b**) Schematic representation of N-terminal and C-terminal Sr-SPIRE protein fragments as used in (a) and their ability to bind (+) or not to bind (-) Sr-MYO5-GTD.

Supplementary Figure 6

raw Western blot images for Figure 4e

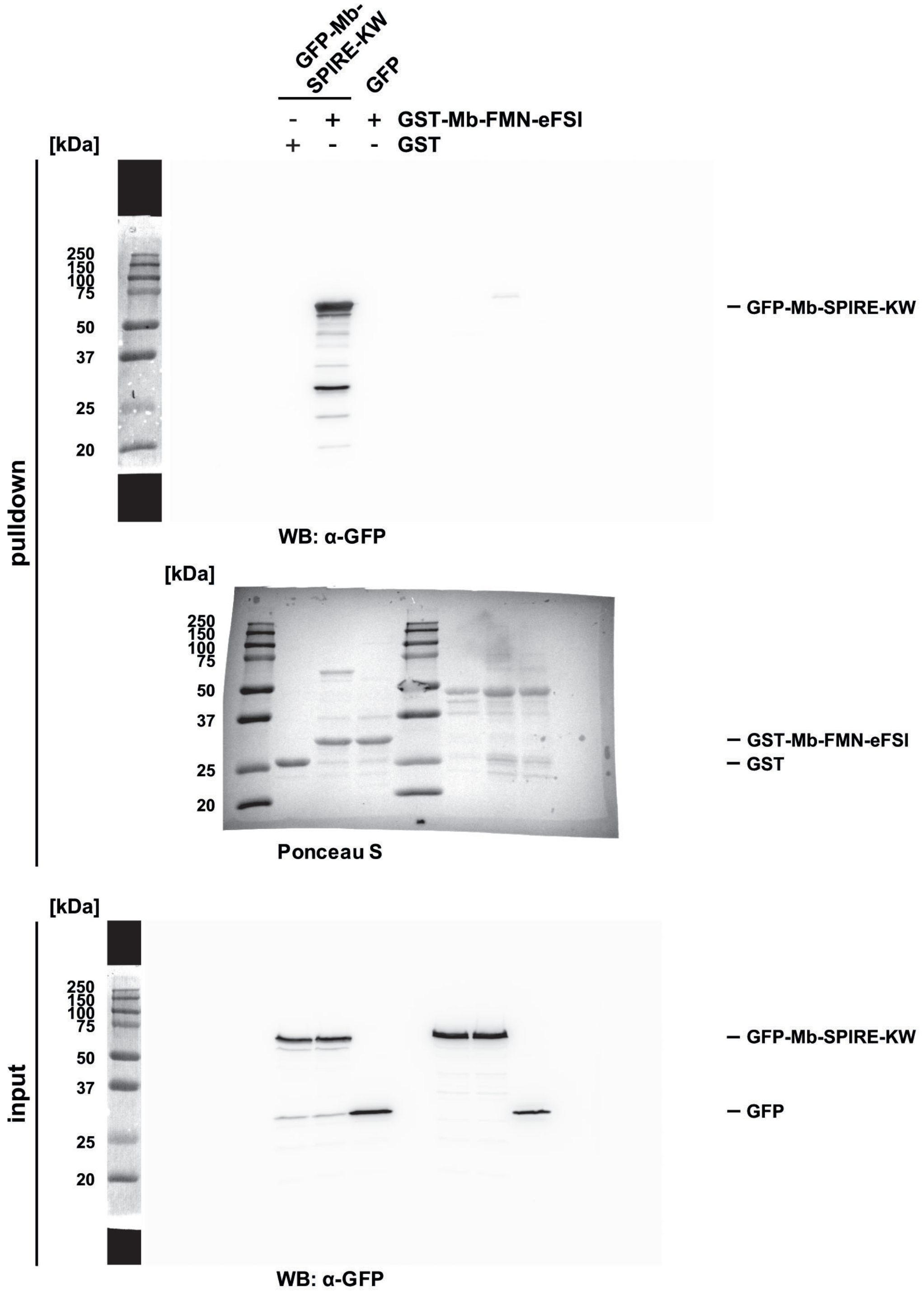

Supplementary Figure 7

raw Western blot images for Figure 5f

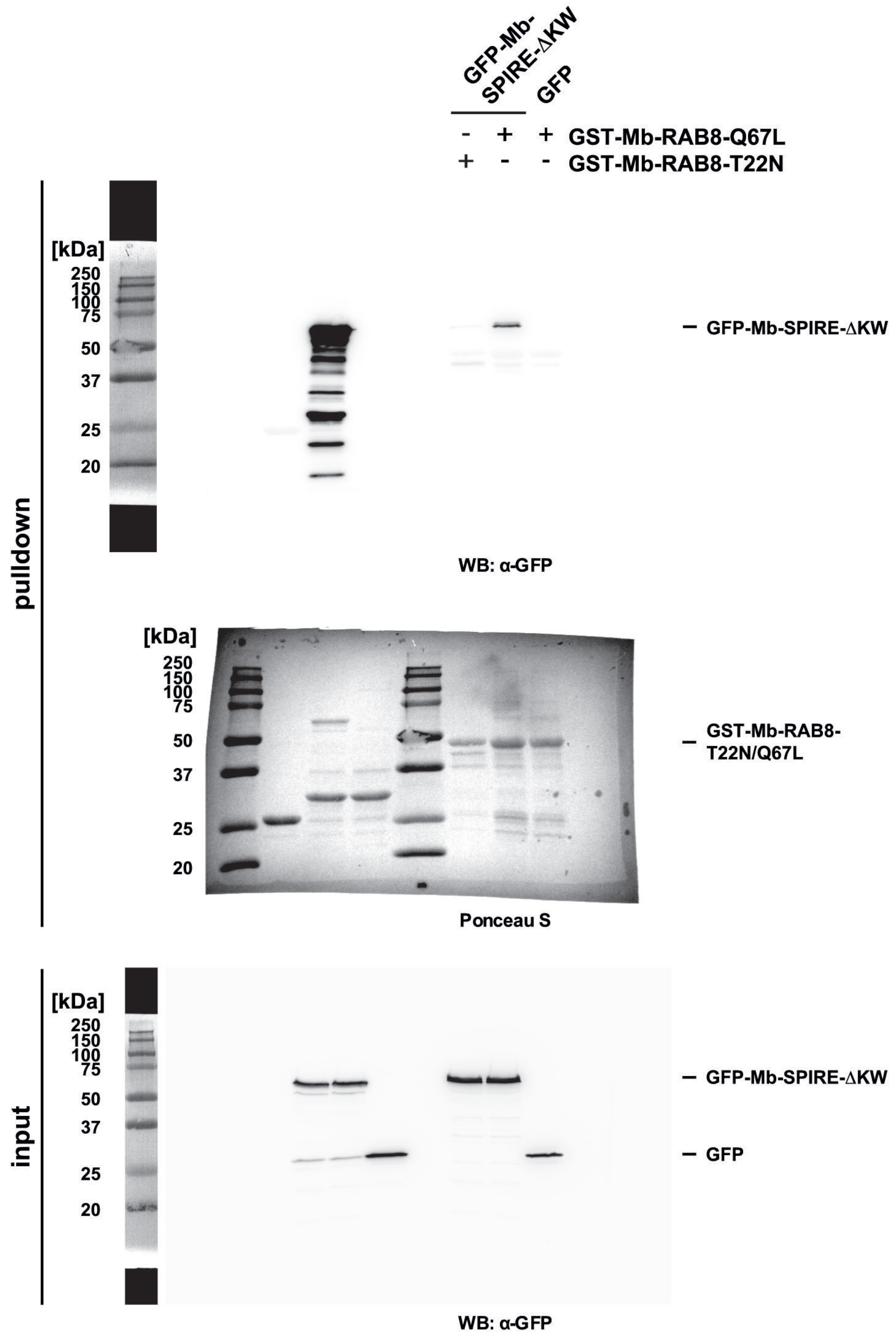

Supplementary Figure 8

raw Western blot images for Figure 6c

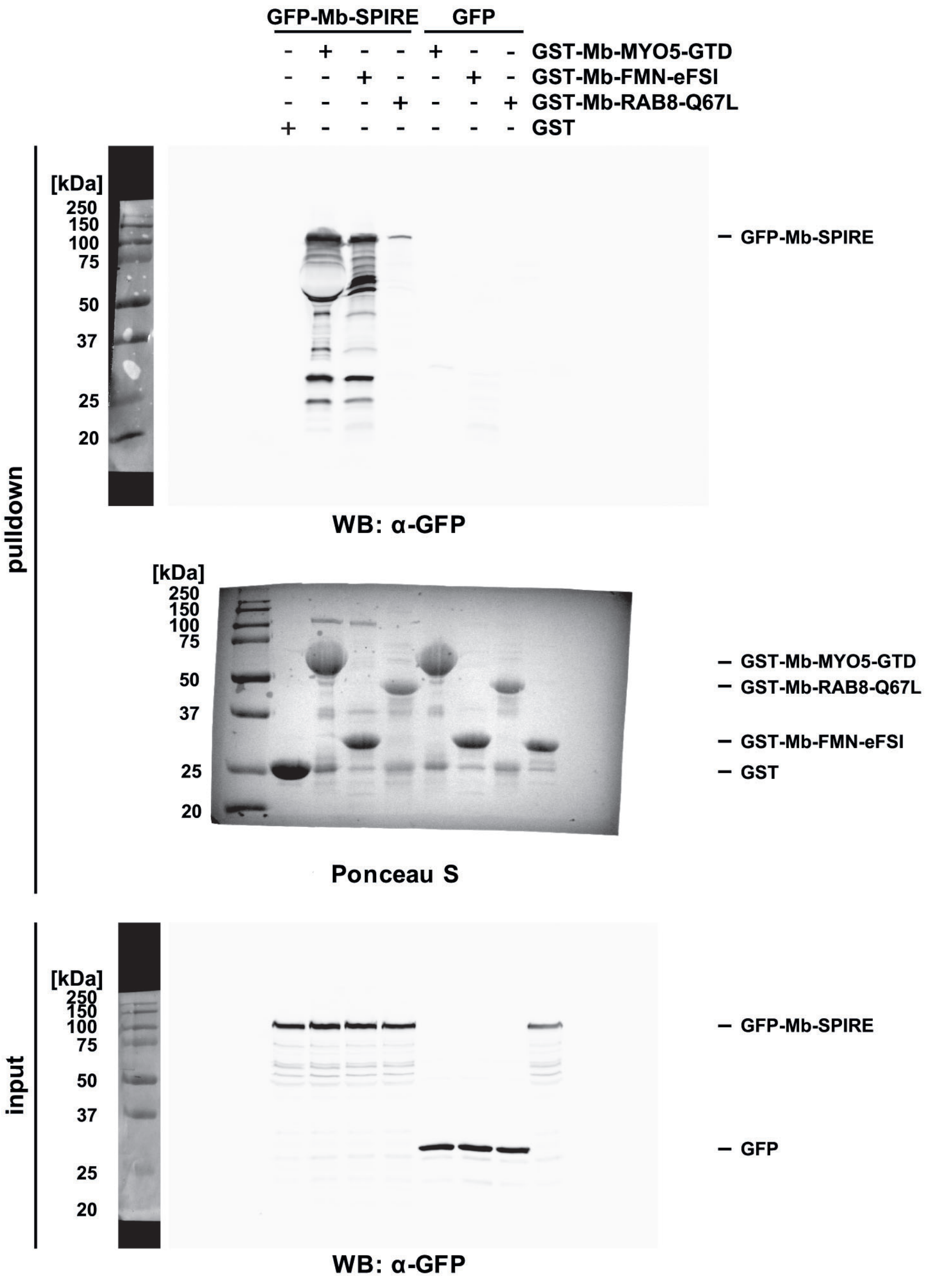

Supplementary Figure 9

raw Western blot images for Figure 6d

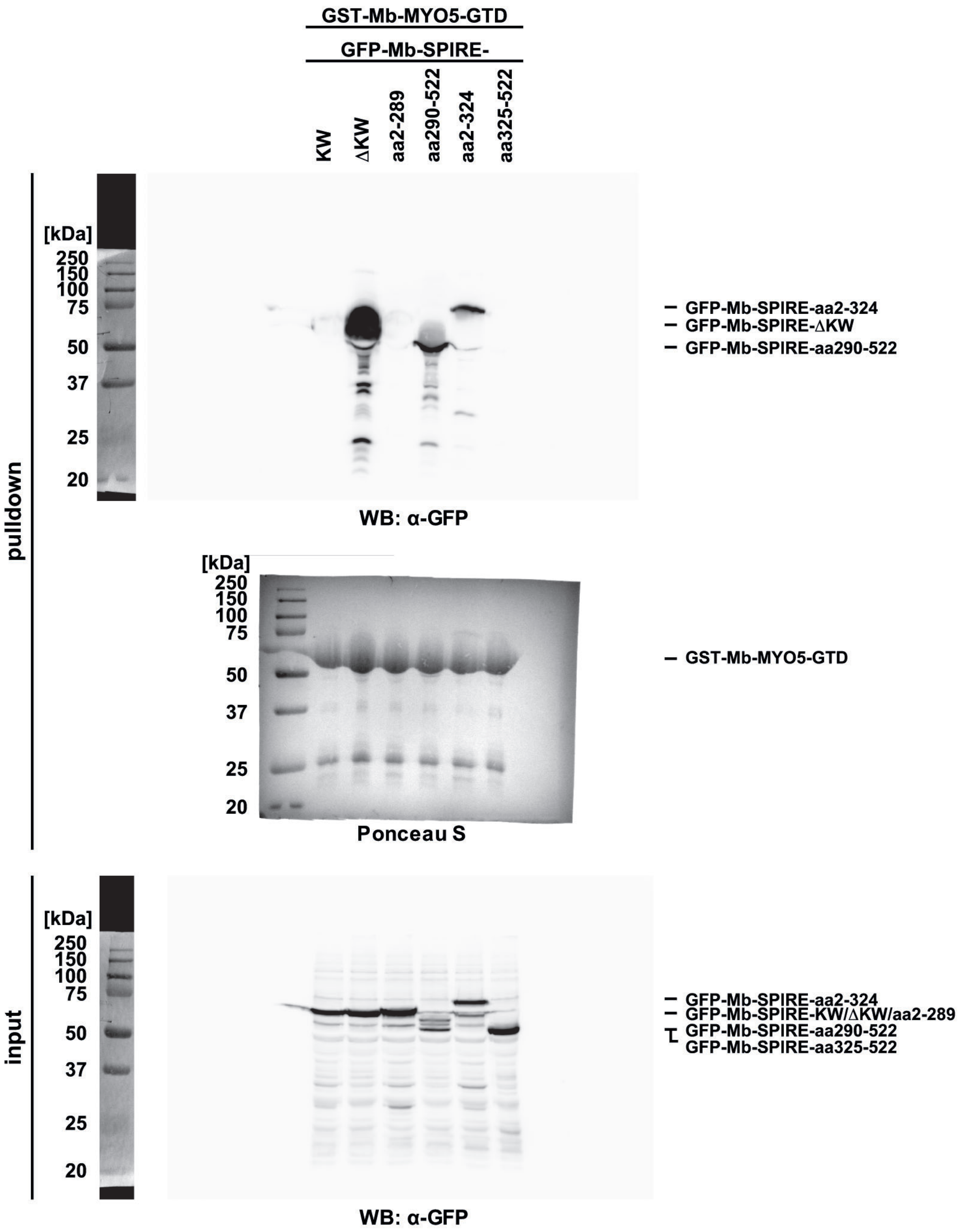

Supplementary Figure 10

raw Western blot images for Figure 8d

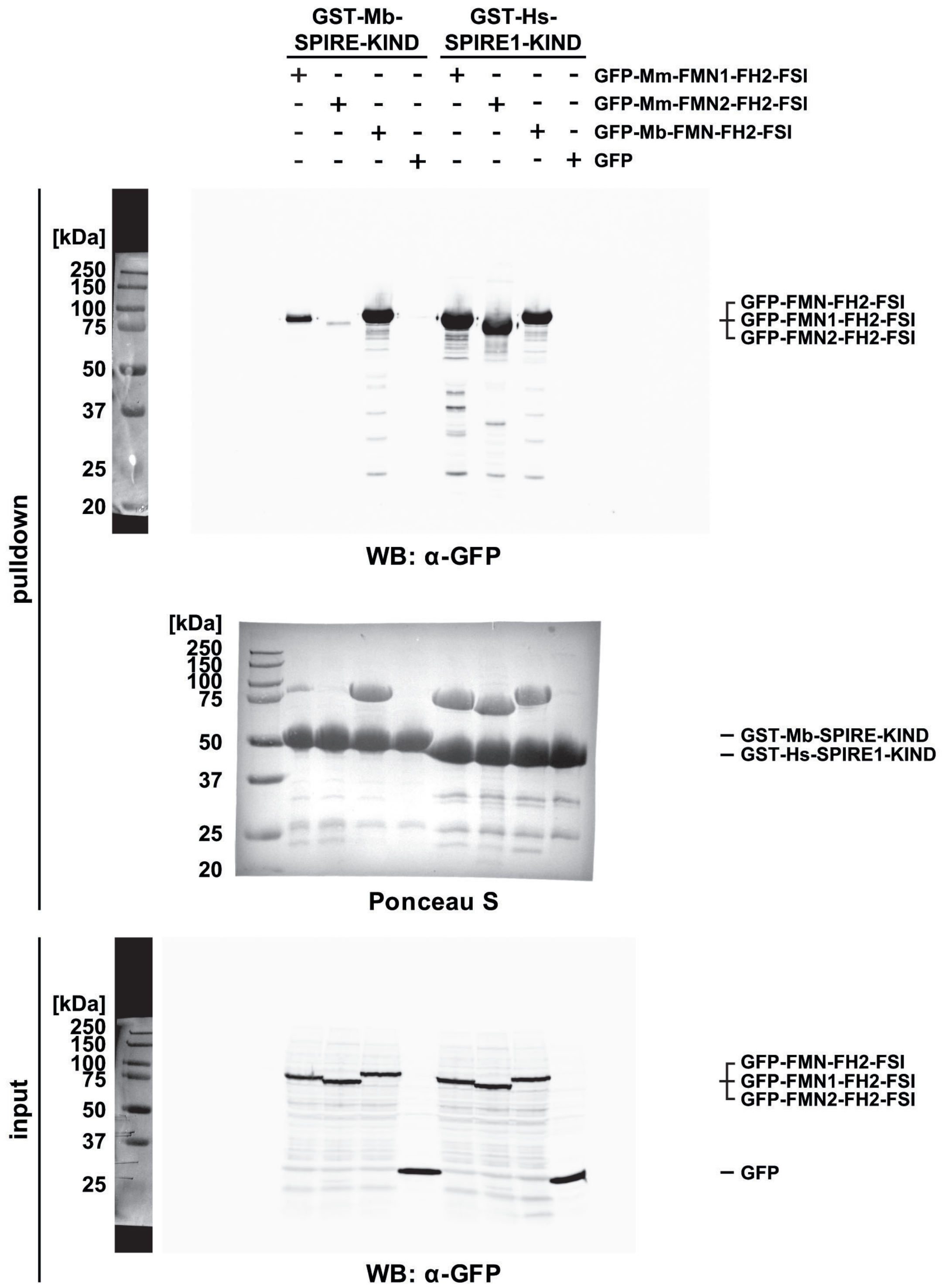

Supplementary Figure 11

raw Coomassie gel images for Supplementary Figure 3

a Mm-SPIRE1

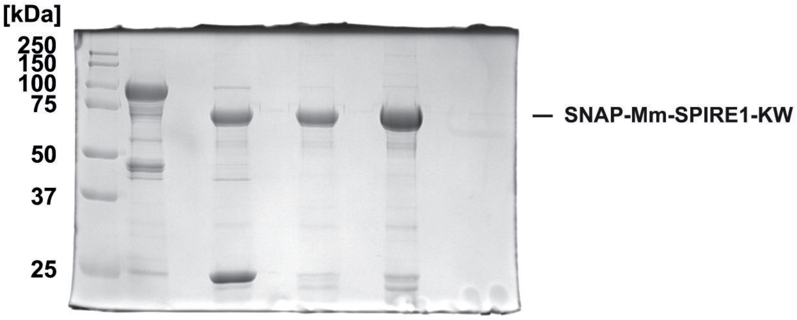

b Mb-SPIRE

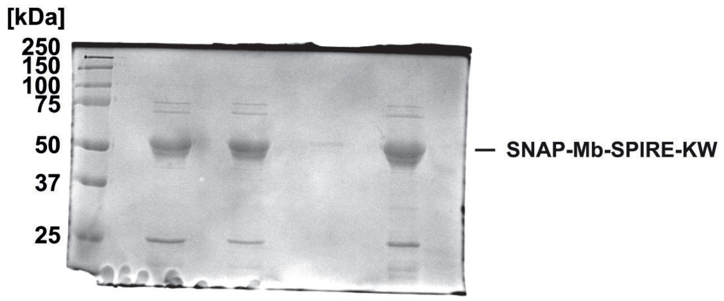

Supplementary Figure 12

raw Western blot images for Supplemenary Figure 5a

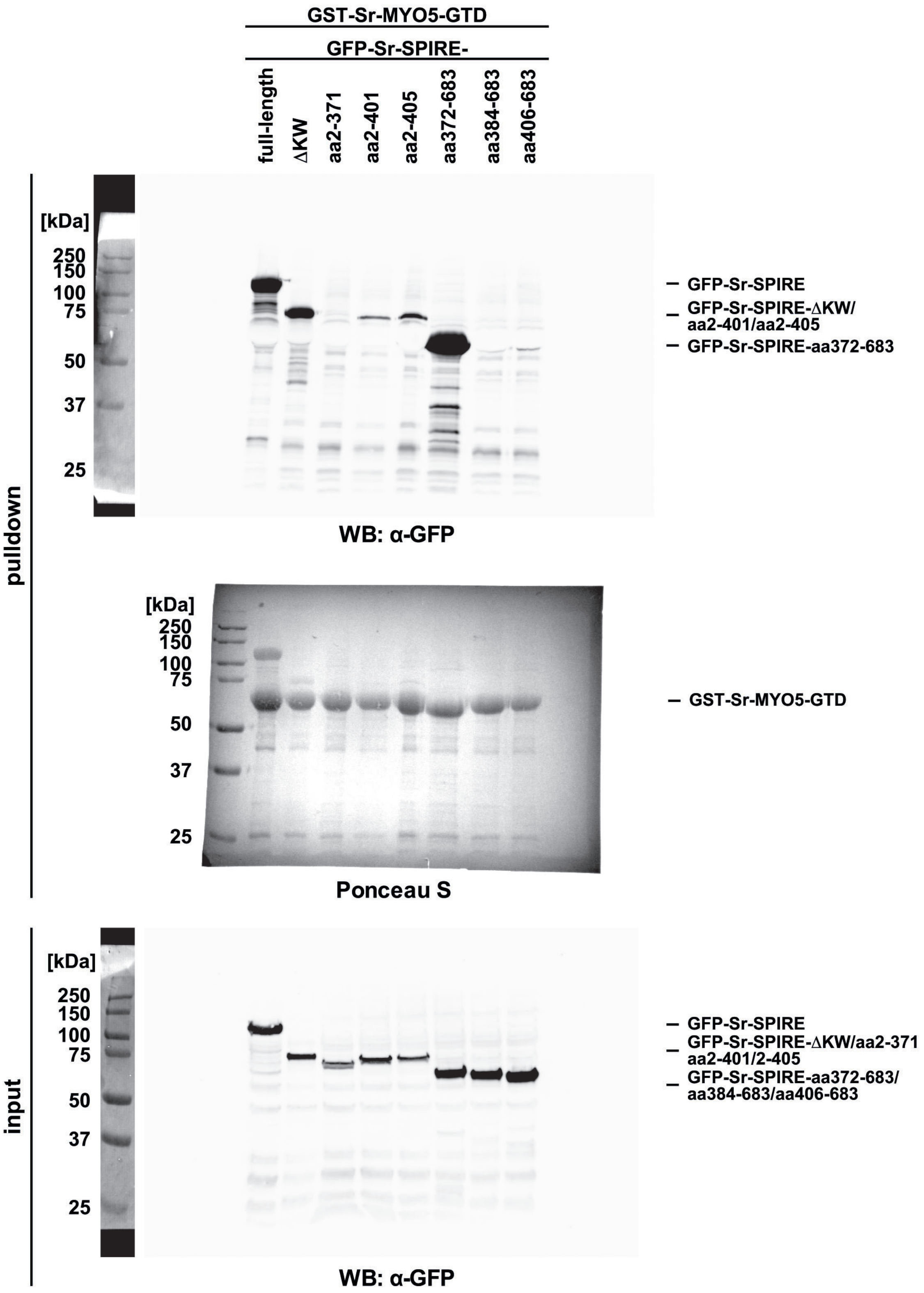

Supplement: Supplementary file 2 — Supplementary information [file 42003_2024_6458_MOESM2_ESM.pdf]
