# Supplementary material for: Actomyosin organelle functions of SPIRE actin nucleators precede animal evolution
Source: Commun Biol. 2024 Jul 8;7:832. doi: 10.1038/s42003-024-06458-1 (PMC11231147; doi:10.1038/s42003-024-06458-1)
Supplement: Supplementary file 3 — Description of Additional Supplementary Materials [file 42003_2024_6458_MOESM3_ESM.pdf]

Description of Additional Supplementary Files

File name: Supplementary Data 1

Description: Numerical Source Data for Figure 2.

File name: Supplementary Data 2

Description: Numerical Source Data for Figure 3b-e.

File name: Supplementary Data 3

Description: Numerical Source Data for Figure 8c.
